# Assessing the Impact of Prolonged Averaging of Coronary Continuous Thermodilution Traces

**DOI:** 10.3390/diagnostics14030285

**Published:** 2024-01-28

**Authors:** Samer Fawaz, Daniel Munhoz, Thabo Mahendiran, Emanuele Gallinoro, Takuya Mizukami, Sarosh A. Khan, Rupert F. G. Simpson, Johan Svanerud, Christopher M. Cook, John R. Davies, Grigoris V. Karamasis, Bernard De Bruyne, Thomas R. Keeble

**Affiliations:** 1 Essex Cardiothoracic Centre, Basildon Hospital, Nether Mayne, Basildon SS16 5NL, UK; 2 Medical Technology Research Centre (MTRC), Anglia-Ruskin University, Chelmsford CM1 1SQ, UK; 3 Cardiovascular Center Aalst, OLV Clinic, 9300 Aalst, Belgium; 4 Department of Advanced Biomedical Sciences, University Federico II, 80138 Naples, Italy; 5 Lausanne University Hospital, 1005 Lausanne, Switzerland; 6 Division of University Cardiology, IRCCS Ospedale Galeazzi Sant’Ambrogio, 20157 Milan, Italy; 7 Division of Clinical Pharmacology, Department of Pharmacology, Showa University, Tokyo 142-0064, Japan; 8 Coroventis Research AB, 756 51 Uppsala, Sweden; 9 School of Medicine, Attikon University Hospital, National and Kapodistrian University of Athens, 157 72 Athens, Greece

**Keywords:** coronary, CMD, continuous thermodilution, microvascular, Coroflow

## Abstract

Continuous Thermodilution is a novel method of quantifying coronary flow (Q) in mL/min. To account for variability of Q within the cardiac cycle, the trace is smoothened with a 2 s moving average filter. This can sometimes be ineffective due to significant heart rate variability, ventricular extrasystoles, and deep inspiration, resulting in a fluctuating temperature trace and ambiguity in the location of the “steady state”. This study aims to assess whether a longer moving average filter would smoothen any fluctuations within the continuous thermodilution traces resulting in improved interpretability and reproducibility on a test–retest basis. Patients with ANOCA underwent repeat continuous thermodilution measurements. Analysis of traces were performed at averages of 10, 15, and 20 s to determine the maximum acceptable average. The maximum acceptable average was subsequently applied as a moving average filter and the traces were re-analysed to assess the practical consequences of a longer moving average. Reproducibility was then assessed and compared to a 2 s moving average. Of the averages tested, only 10 s met the criteria for acceptance. When the data was reanalysed with a 10 s moving average filter, there was no significant improvement in reproducibility, however, it resulted in a 12% diagnostic mismatch. Applying a longer moving average filter to continuous thermodilution data does not improve reproducibility. Furthermore, it results in a loss of fidelity on the traces, and a 12% diagnostic mismatch. Overall, current practice should be maintained.

## 1. Introduction

The invasive assessment of coronary microvascular dysfunction (CMD) is a growing field, particularly following the success of the CorMiCa trial (Coronary Microvascular Angina trial, NCT: NCT03193294), showing that an invasive diagnostic procedural approach resulted in improved symptoms and quality of life for patients with angina with non-obstructive coronary artery disease (ANOCA) [1]. Additionally, a recent randomised control trial by Sinha et al., showed that ANOCA patients with a reduced coronary flow reserve (CFR) benefit from anti-ischaemic therapy [2]. Moreover, a meta-analysis and systematic review by Kelshiker et al. has shown that CFR holds significant prognostic value across a wide range of cardiac pathologies [3].

In contemporary practice, several methodologies for the invasive evaluation of CMD exist [4], namely, Doppler [5,6], Bolus Thermodilution [7,8], Continuous Thermodilution [9,10], and angiogram-derived [11,12,13]. The various benefits, pitfalls, and differences between them have been the subject matter of several notable publications [14,15,16,17,18].

Continuous Thermodilution is a novel method in the invasive assessment of coronary microvascular dysfunction (CMD) which allows the volumetric quantification of absolute coronary flow (Q) in mL/min from which subsequent microvascular indices can be derived. It has been demonstrated to be safe and operator independent in multiple studies [19,20]. Additionally, it has demonstrated good correlations with invasive Doppler assessment [21], as well as non-invasive ^15^O-H_2_O-PET assessment [17]. Furthermore, it has superior reproducibility to the other widely utilised method of bolus thermodilution [15].

However, it is not without pitfalls. During the continuous thermodilution procedure, temperature is measured continuously during the intracoronary saline infusion at a sampling rate of 100 Hz allowing the quantification of Q throughout the cardiac cycle. By default, the proprietary analysis software “Coroflow” (Version 3.4, Coroventis, Uppsala, Sweden), applies a 2 s moving average filter to the temperature trace to average out the significant variation in flow throughout the cardiac cycle. The moving average filter can also be increased to 4 or 6 s from within the software settings. This, however, can be ineffective in the presence of irregular heart rates, ventricular extrasystoles, and deep inspiration, resulting in unsteady traces, ambiguity regarding the placement of markers denoting distal mixed temperature (T) and infusate temperature (T_i_), and a theoretical under or over estimation of Q.

**Scheme 1:**
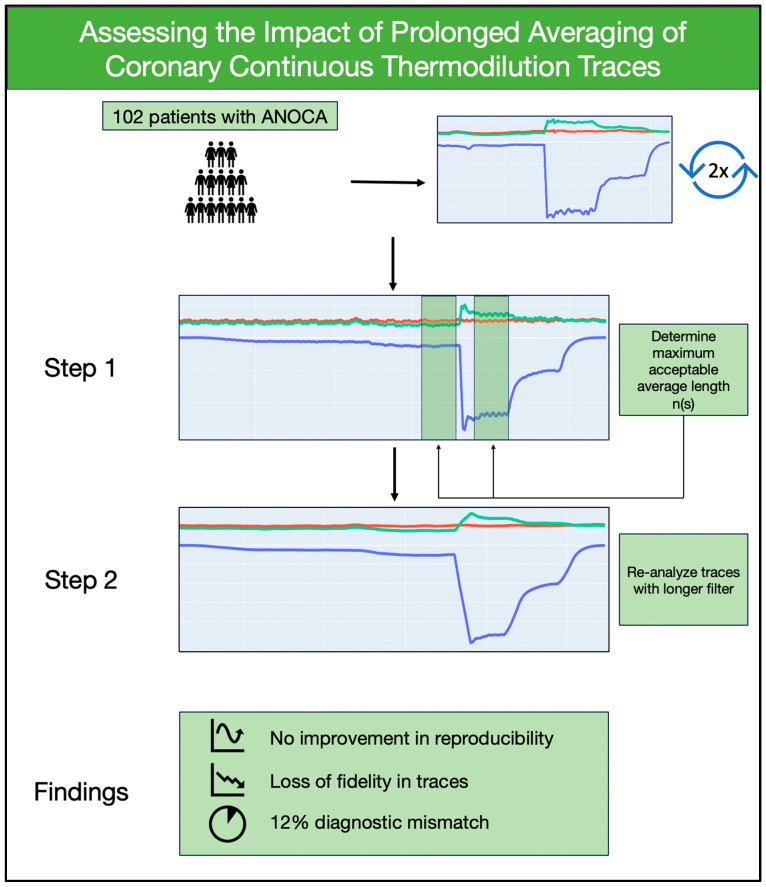
Experimental Assessment of Longer Continuous Thermodilution Moving Average Filter. Temperature trace (blue), P_d_ trace (green), P_a_ trace (red).

This study aims to investigate whether using a longer moving average filter will result in improved reproducibility whilst maintaining diagnostic agreements and accuracy (Scheme 1). Additionally, whether a longer filter will adequately smoothen the data, in order to improve interpretability.

## 2. Methods

### 2.1. Study Design

This is a sub-analysis of a previously reported study [15]. Patients with angina and non-obstructive coronary artery disease (ANOCA) were prospectively enrolled from January 2021 to January 2022. The study protocol was approved by the institutional review board (IRB of the Onze-Lieve-Vrouw Clinic in Aalst, Belgium, Registration number: 2020/033). The full design and results of the parent study can be found published in EuroIntervention [15].

### 2.2. Coronary Angiography

Coronary angiography was performed via either the radial or femoral route using 6Fr guiding catheters. Isosorbide Dinitrate (0.2 mg) was administered intracoronary. A specialised coronary guidewire with a distal pressure and temperature sensor was advanced to the equalization position, and the aortic pressure (P_a_) and distal coronary pressure (P_d_) were equalized on the dedicated console (Coroflow, Coroventis, Uppsala, Sweden). The coronary guidewire was then advanced into the coronary to a distal position.

### 2.3. Continuous Thermodilution Measurements

Continuous thermodilution measurements were performed according to previously described methodology [22]. A dedicated monorail 2.52Fr infusion microcatheter with four distal side holes (Rayflow, Hexacath, Paris, France) [23] was connected to an automatic infusion pump and flushed outside the body with room temperature saline at a rate of between 10 and 20 mL/min. It was subsequently advanced over the specialised coronary guidewire and advanced to the first few millimetres of the coronary artery under investigation. A continuous recording of aortic pressure (P_a_), distal coronary pressure (P_d_) and Temperature was performed during infusion of room temperature saline at rates of 10 mL/min (resting) followed by 20 mL/min (hyperemic) until steady state was achieved [24]. Following this, the coronary guidewire was swiftly pulled back until the pressure/thermistor segment was within the Rayflow catheter to record the temperature of the infusate (T_i_) at 20 mL/min followed by 10 mL/min. This process was then repeated once again in each patient to assess reproducibility.

### 2.4. Analysis of Traces

Continuous thermodilution traces were extracted from Coroflow into .csv format. A dedicated app was programmed using Python (Version 3.10.9, Python Software Foundation, Wilmington, DE, USA) with Plotly library (Plotly, Montreal, QC, Canada), that facilitated plotting of continuous thermodilution traces. The data was analysed in two stages.

In the first stage, the temperature trace was plotted using a 2 s moving average as is default in Coroflow. When a data point was clicked within the dedicated app, simultaneous 2,10,15, and 20 s averages of the P_d_, P_a_, and raw temperature up to that datapoint were automatically calculated and displayed within a table in the app. Accordingly, the analysis of each trace required four clicks: a click each within the usual “steady state” period of T and T_i_, repeated at both saline infusion rates. For the purposes of this study, a 2 s average of data was considered the reference standard, as Coroflow defaults to a 2 s moving average. This analysis was used to determine the maximum acceptable average (*see Section 2.7: Determination of Maximum Length of Average*).

**Figure 1:**
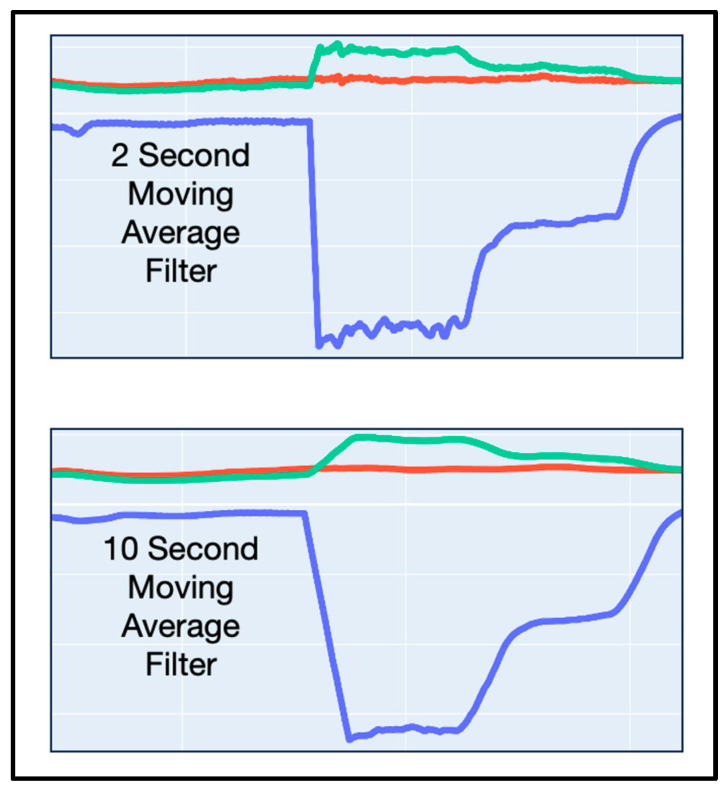
Demonstration of continuous thermodilution traces visualised with a 2 s moving average (**above**), and 10 s moving average (**below**). Temperature trace (blue), P_d_ trace (green), P_a_ trace (red).

In the second stage, the data was re-plotted using a longer moving average filter as determined in the first stage. The data was then reanalysed in its entirety with the longer moving average filter in order to simulate the real-world consequences of lengthened smoothening of data (Figure 1).

### 2.5. Definition of a Moving Average Filter

A moving average filter is a data smoothing method that is used to minimise noise and emphasise patterns in a dataset. It works by taking the mean of a window size (a predetermined number of consecutive data points) and advancing this window over the dataset. A new set of data points is produced as a result, each of which represents the average value of the original data during a particular period of time. The resulting plots display less fluctuations and variability resulting in a smoother curve.

### 2.6. Calculation of Absolute Flow (Q)

The calculations of *Q* at different length averages were calculated as:

$$Q=\frac{T_{i}}{T}*1.08*Q_{i}$$

where “*Q*” is absolute flow, “*T_i_*” is the infusate temperature, “*T*” is the distal mixed temperature, and “*Q_i_*” represents the infusion rate of saline.

The remaining formulas used for the calculation of continuous thermodilution indices are supplied in the supplemental material (Supplementary Table S1). All indices were calculated using the corresponding averages of *T_i_*, *T*, P_d_, and P_a_.

### 2.7. Determination of Maximum Length of Average

To ascertain the maximum acceptable length of averaged data, it was determined that each tested average must meet the following criteria when compared to the reference standard of a 2-s average: the Q (rest and hyperemic) values should have a mean bias no greater than ±5 mL/min. Hyperemic Microvascular Resistance (R_µ,hyper_) should have a mean bias no greater than ±5 Woods Units (WU). CFR should have a mean bias no greater than ±0.1 CFR points. MRR should have mean bias no greater than ±0.1 MRR points. Additionally, it was mandated that the longer averages display a diagnostic accuracy (*see Section 2.8: Statistical Analysis*) of ≥90% for CFR (cut off < 2.5) and R_µ,hyper_ (cut off > 400) when compared to 2 s average as a reference standard.

### 2.8. Statistical Analysis

The distribution of continuous variables was assessed visually using Q-Q plots and histograms. Continuous data are expressed as mean (±standard deviation), or median (25th–75th percentile) as appropriate. Mean bias is expressed as Mean (95% Confidence Intervals). Correlations were assessed using Pearson’s r. The Intraclass Correlation Coefficient (ICC) was calculated using a single measure, two-way mixed effect model with measures of absolute agreement. Bland–Altman analysis was used to quantify the agreement between repeated measurements. Diagnostic accuracy was calculated using the formula:

$$Accuracy=\frac{TP+TN}{TP+TN+FP+FN}$$

where “*TP*” = true positive, “*TN*” = true negative, “*FP*” = false positive, “*FN*” = false negative. Variability was calculated as a relative difference of two measurements (*A* and *B*) and expressed as a percentage according to the formula:

$$Var\left(\%\right)=\frac{A-B}{(A+B)/2}*100$$

All analyses were performed using SPSS (IBM, New York, NY, USA). *A p* value of <0.05 was considered statistically significant.

## 3. Results

### 3.1. Baseline and Procedural Data

All 102 patients included in the parent study were included in this sub-analysis. No traces were excluded on the basis of insufficient steady state periods. The full baseline and procedural data can be viewed as published in EuroIntervention [15].

### 3.2. Determination of Maximum Acceptable Average

With pooled test and re-test data of the three averages tested (10, 15 and 20 s), only the average of 10 s fulfilled all the criteria to be acceptable as a longer average (Table 1). Compared to the reference standard, the mean bias of Q_rest_ was −1.02 (95% CI −1.66–−0.39) mL/min, the mean bias of Q_hyper_ was 2.71 (95% CI 0.91–4.51) mL/min, the mean bias of CFR was 0.07 (95% CI 0.05–0.10), the mean bias of MRR was 0.1 (95% CI 0.06–0.13), and the mean bias of R_µ,hyper_ was −4.86 (95% CI −7.86–−1.87) WU (Table 1). All indices at 10 s averages were highly correlated with their reference standard counterpart (r = 0.97–0.99, *p* < 0.001 for all) (Supplementary Figure S1).

Similarly, of the three averages tested, only the average of 10 s fulfilled the diagnostic accuracy criteria, with a CFR accuracy of 90%, and a R_µ,hyper_ accuracy of 95%. The complete list of accuracies of each average CFR and R_µ,hyper_ is located within Table 1**.**

### 3.3. Re-Analysis with a 10 s Moving Average Filter

Following re-analysis of the traces with a 10 s moving average filter applied, the mean bias from the combined data (test and re-test) of indices calculated with a 10 s moving average were compared to indices calculated with a 2 s average as a reference standard. The mean bias of Q_rest_ was 1.81 (95% CI 1.02–2.59) mL/min, the mean bias of Q_hyper_ was −2.47 (95% CI −4.53–−0.41) mL/min, the mean bias of CFR was −0.09 (95% CI −0.12–−0.06), the mean bias of MRR was −0.11 (95% CI −0.15–−0.07), and the mean bias of R_µ_,_hyper_ was 4.08 (95% CI 0.22–7.95) WU (Supplementary Table S2). All indices derived using a 10 s moving average filter were highly correlated with their reference standard counterparts (r = 0.96–0.98, *p* < 0.001) (Supplementary Table S2). With a 10 s moving average filter applied, the diagnostic accuracy of CFR was 89% (CFR < 2.5), and R_µ_,_hyper_ was 94% (R_µ,hyper_ > 400), when compared to the reference standard.

### 3.4. Assessment of Reproducibility

**Figure 2:**
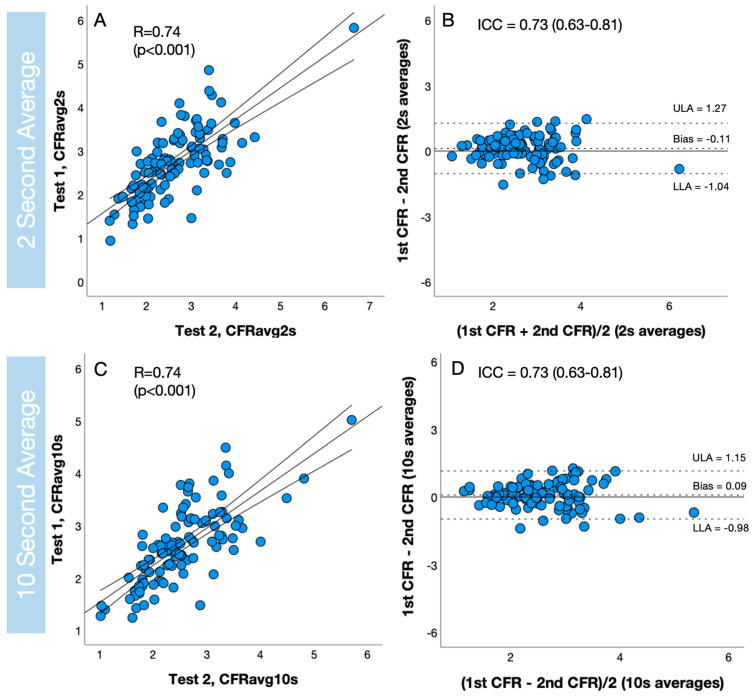
Reproducibility of CFR_._ Scatter plot of first and second CFR measurement using 2 s average (**A**), and associated Bland–Altman plot (**B**). Scatter plot of first and second CFR measurement using 10 s average (**C**), and associated Bland–Altman plot (**D**). “CFR” = Coronary Flow Reserve, “R” = Pearson’s r, “ICC” = Intraclass Correlation Coefficient.

**Figure 3:**
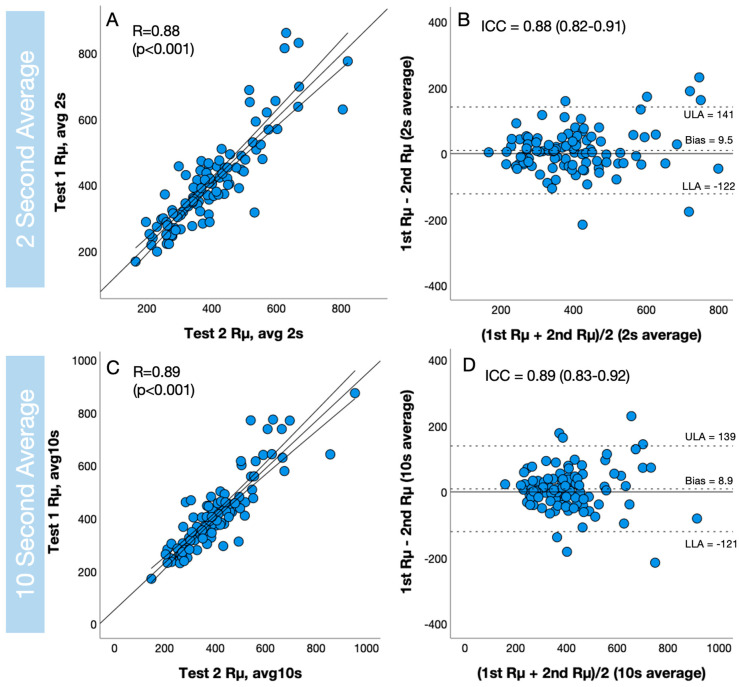
Reproducibility of R_µ,hyper_. Scatter plot of first and second R_µ,hyper_ measurement using 2 s average (**A**), and associated Bland–Altman plot (**B**). Scatter plot of first and second R_µ,hyper_ measurement using 10 s average (**C**), and associated Bland–Altman plot (**D**). “R_µ,hyper_” = Hyperemic Microvascular Resistance, “R” = Pearson’s r, “ICC” = Intraclass Correlation Coefficient.

The reproducibility data from duplicate measurements of continuous thermodilution derived indices using a 10 s moving average filter, as well as continuous thermodilution derived indices using a 2 s average are displayed in Table 2. Overall, there was no significant improvement in reproducibility when a 10 s moving average filter was applied, however, no significant deterioration was observed. Figure 2 and Figure 3, illustrate the individual correlations, scatter plots, and Bland–Altman analyses for repeated measurements of CFR and R_µ,hyper_, respectively, analysed using both a 2 s and 10 s moving average filter.

## 4. Discussion

### 4.1. Summary of Results

This study is the first of its kind to examine the accuracy and reproducibility of alternative methods of interpreting raw invasive pressure/thermistor data acquired during continuous thermodilution measurements. We tested the hypothesis that a longer moving average filter would smoothen any irregularities within the continuous thermodilution traces resulting in less variation in measurements on a test–retest basis. Conclusively, we have not demonstrated superiority of a longer moving average filter for three main reasons: Firstly, there was no demonstrated improvement in reproducibility, variability (%), or correlation of test–retest when compared to the reference standard. Secondly, when a longer 10 s moving average filter was applied, there was a loss of fidelity in the temperature traces. Lastly, a clinically unacceptable level of diagnostic mismatch occurs when a longer filter is applied.

### 4.2. Reproducibility

There was no demonstrated improvement in reproducibility noted after re-analysis with a 10 s moving average filter. Of note, during the determination of the longest acceptable average, there was a slight numerical improvement in some of the reproducibility metrics (Supplementary Table S3); however, at this stage of the analysis, the temperature data was plotted using the standard 2 s moving average filter (see first stage of the analysis of traces) allowing the “steady state” portions of the traces to be identified easily. When a 10 s moving average filter is applied, a short “steady state” period can be lost within the slope of the temperature curve and any appreciable benefit is lost.

### 4.3. Loss of Fidelity with 10 s Moving Average

A 10 s moving average filter results in the smoothening of any peaks or troughs caused by variations in flow due to irregular heart rhythms, ventricular extrasystoles, or deep inspiration. However, as we have noted during analysis, this also results in a loss of fidelity and inadvertent loss of crucial sections of the trace.

In several of the analysed traces, the “steady state” period during T_i_ measurement can be brief, as there is often a phenomenon of irregular temperature traces noted immediately after the wire is pulled back into the Rayflow catheter. This phenomenon is likely to be caused by sudden pressure changes related to the proximity of the coronary guidewire pressure transducer to the Rayflow catheter spray, as noted in the P_d_ trace which climbs to supraphysiological levels during this portion of the measurement. Normally, this irregular temperature trace takes around 10–15 s to re-settle following the pullback manoeuvre, before slowly reaching a steady state from which the T_i_ measurement can be selected. If the steady state period is short, it can be lost when a 10 s moving average is applied. Moreover, another portion of the T_i_ phase can subsequently appear to be longer and more stable therefore shifting the position of T_i_ measurement entirely. As a result, the traces become harder to interpret, rather than easier. Of note, this is not a perceivable issue during the T portion of the measurement.

### 4.4. Diagnostic Mismatch

**Figure 4:**
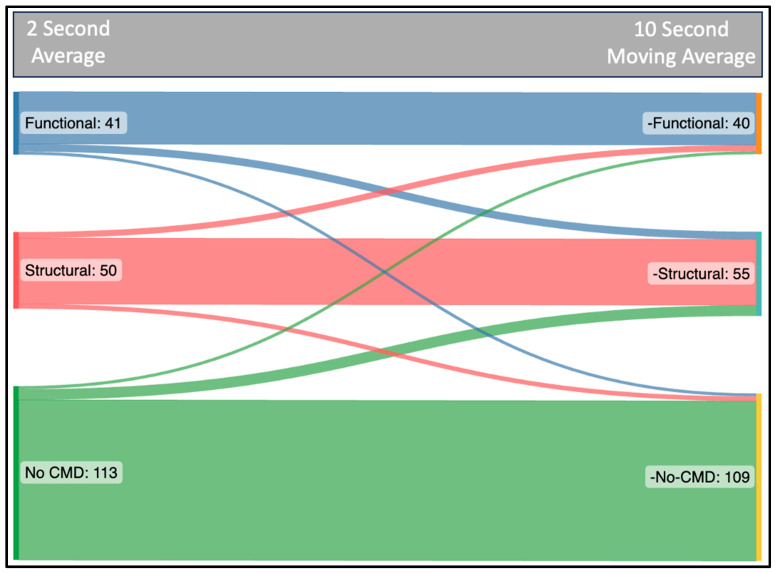
Sankey chart of microvascular phenotypes classification. Classified on left using reference standard 2 s average, followed by direction of classification using 10 s moving average filter applied.

When a 10 s moving average is applied, a numerically respectable diagnostic accuracy (CFR accuracy 89%, R_µ,hyper_ accuracy 94%) is achieved; however, when considered from a clinical standpoint, it reflects an unacceptable level of diagnostic mismatch. To illustrate further, when microvascular phenotypes (structural or functional, versus no CMD) are determined, there is a 12% diagnostic mismatch when a 10 s moving average filter is applied (Figure 4).

## 5. Limitations

There are several limitations. First, this is a retrospective sub-analysis; however, the original intention for the data, as well as its quality, rendered it ideal for this analysis. Second, as these were high quality data, we cannot rule out the possibility of this method working for lower quality data. Third, the trace analysis was performed by a single unblinded assessor which may introduce bias. This was mitigated by analysing all the first test traces consecutively and uninterrupted, before moving to the second test traces using a separate datasheet. Finally, a dedicated app was encoded for the purposes of the analysis, and although unlikely, there may be small differences in how the native Coroflow software handles the data.

## 6. Conclusions

Applying a longer moving average of 10 s to the continuous thermodilution traces has a negligible effect on reproducibility. Furthermore, it has a deleterious effect on the interpretability of the traces due to loss of fidelity. Lastly, it results in unacceptable levels of diagnostic mismatch relative to the reference standard. As a result, it is advisable to maintain the current practice. Notwithstanding, offering comprehensive guidance on cursor placement during trace analysis could prove beneficial for inexperienced users. Accordingly, a standardisation of continuous thermodilution methodology would be a welcome addition to the literature base.

## Figures and Tables

**Table 1:** Determination of maximum acceptable averages of continuous thermodilution indices. Acceptable (green), not acceptable (red).

	2 s Average (Reference) Mean ± SD	10 s Average	15 s Average	20 s Average
		Mean Bias (95% CI)	Mean Bias (95% CI)	Mean Bias (95% CI)
Q_rest_	82 ± 30 mL/min	−1.02 (−1.66–−0.39)	−1.76 (−2.52–−1.00)	−2.71 (−3.59–−1.84)
Q_hyper_	211 ± 77 mL/min	2.71 (0.91–4.51)	4.03 (1.80–6.26)	6.13 (3.04–9.23)
R_µ,hyper_	403 ± 135 WU	−4.86 (−7.86–−1.87)	−6.56 (−10.63–−2.5)	−11.3 (−17.18–−5.08)
CFR	2.67 ± 0.81	0.07 (0.05–0.10)	0.12 (0.08–0.15)	0.17 (0.13–0.21)
MRR	3.24 ± 0.96	0.10 (0.06–0.13)	0.13 (0.09–0.17)	0.20 (0.15–0.25)
**Accuracy (%)**				
CFR (<2.5)		90%	87%	84%
R_µ,hyper_ (>400)		95%	92%	90%

“Q_rest_” = Resting coronary flow, “Q_hyper_” = Hyperemic absolute coronary flow, “R_µ,hyper_” = Hyperemic microvascular resistance, “CFR” = Coronary Flow Reserve, “MRR” = Microvascular Resistance Reserve.

**Table 2:** Means ± SD of Test 1 and Test 2 continuous thermodilution indices analysed with 2 and 10 s averages. Comparison of test–retest reproducibility of a 2 s average (reference), versus a 10 s moving average filter. “CFR” = Coronary Flow Reserve, “MRR” = Microvascular resistance reserve, “R_µ_,_hyper_” = Hyperemic Microvascular Resistance, “Q_rest_” = resting absolute flow, “Q_hyper_” = hyperemic absolute flow. “R” = Pearson’s r, “ICC” = Intraclass Correlation Coefficient. “Var (%)” = Variability Percentage, “CI” = Confidence interval.

	2 s Average					10 s Moving Average Filter				
	Test 1	Test 2	R (95% CI)	ICC (95% CI)	Var (%)	Test 1	Test 2	R (95% CI)	ICC (95% CI)	Var (%)
CFR	2.73 ± 0.80	2.61 ± 0.82	0.74 (0.63–0.81)	0.73 (0.62–0.81)	17.8 ± 12.4	2.62 ± 0.73	2.54 ± 0.76	0.74 (0.63–0.81)	0.73 (0.63–0.81)	16.2 ± 12.1
MRR	3.27 ± 0.95	3.22 ± 0.97	0.72 (0.61–0.80)	0.72 (0.61–0.80)	16.5 ± 12.4	3.16 ± 0.89	3.11 ± 0.91	0.73 (0.63–0.81)	0.73 (0.63–0.81)	16.2 ± 11.8
R_µ,hyper_	408 ± 142	398 ± 129	0.88 (0.83–0.92)	0.88 (0.82–0.91)	11.9 ± 9.9	412 ± 139	403 ± 137	0.89 (0.83–0.92)	0.89 (0.83–0.92)	11.4 ± 10
Q_rest_	79 ± 28	86 ± 32	0.73 (0.62–0.81)	0.70 (0.57–0.79)	20.3 ± 16.0	80 ± 28	88 ± 34	0.77 (0.76–0.84)	0.73 (0.59–0.82)	20 ± 14.8
Q_hyper_	207 ± 73	216 ± 81	0.87 (0.81–0.91)	0.86 (0.80–0.90)	12.5 ± 11.1	202 ± 67	215 ± 81	0.89 (0.84–0.92)	0.86 (0.79–0.91)	13 ± 11.1

## Data Availability

The data presented in this study are available in the main article and Supplementary Materials.

## Supplementary Materials

The following supporting information can be downloaded at: https://www.mdpi.com/article/10.3390/diagnostics14030285/s1, Figure S1. Scatter plots of 2 second average indices (*x*-axis), against 10 second average indices (*y*-axis). A = Qrest, B = Qhyper, C = Rµ,hyper, D = CFR, E = MRR; Table S1. Formulas for the calculation of Continuous Thermodilution indices, Table S2. Continuous thermodilution indices derived with a 10 second moving average filter, compared to 2 seconds average as a reference standard, Table S3. Reproducibility data for continuous thermodilution derived indices at 2 and 10 second averages. Traces visualised with the default 2 second moving average filter for both.

## Abbreviations

**ANOCA**	Angina with Non-Obstructive Coronary Artery Disease
**CFR**	Coronary Flow Reserve
**CMD**	Coronary Microvascular Dysfunction
**FFR**	Fractional Flow Reserve
**MRR**	Microvascular Resistance Reserve
**P_a_**	Aortic pressure
**P_a,rest_**	Aortic pressure at rest (mmHg)
**P_a,hyper_**	Aortic pressure at maximum coronary hyperemia (mmHg)
**P_d_**	Distal coronary pressure
**P_d,rest_**	Distal coronary pressure at rest (mmHg)
**P_d,hyper_**	Distal coronary pressure at maximum coronary hyperemia (mmHg)
**PET**	Positron Emission Tomography
**Q**	Absolute Coronary Flow (mL/min)
**Q_rest_**	Absolute Coronary Flow at rest (mL/min)
**Q_hyper_**	Absolute Coronary Flow during hyperemia (mL/min)
**Q_i_**	Saline Infusion Rate
**R_µ,hyper_**	Hyperemic Microvascular Resistance
**WU**	Woods Units
**T**	Distal Coronary Mixed Temperature
**T_i_**	Difference between blood temperature and the temperature of saline at the exit of the infusion catheter (°C)
