# Supplementary material for: Assessing the Impact of Prolonged Averaging of Coronary Continuous Thermodilution Traces
Source: Diagnostics (Basel). 2024 Jan 28;14(3):285. doi: 10.3390/diagnostics14030285 (PMC10855808; doi:10.3390/diagnostics14030285)
Supplement: Supplementary file 1 [file diagnostics-14-00285-s001.zip › diagnostics-2524946-supplementary.pdf]

Supplemental Materials

Supplementary Table S1. *Formulas for the calculation of Continuous Thermodilution indices*

Index	Formula
FFR	$\frac{P_{d,hyper}}{P_{a,hyper}}$
$R_{\mu,hyper}$	$\frac{P_{d,hyper}}{Q_{hyper}}$
CFR	$\frac{Q_{hyper}}{Q_{rest}}$
MRR	$\frac{CFR}{FFR} * \frac{P_{a,rest}}{P_{a,hyper}}$

Where "FFR" = Fractional Flow Reserve, "$R_{\mu,hyper}$" = Hyperemic Microvascular Resistance, "CFR" = Coronary Flow Reserve, "MRR" = Microvascular Resistance Reserve, "P_d" = Distal Coronary Pressure, "P_a" = Aortic Pressure, "Q" = Absolute Flow, "rest" = resting state, "hyper"= hyperemic state.

Supplementary Table S2. Continuous thermodilution indices derived with a 10 second moving average filter, compared to 2 seconds average as a reference standard.

	10 Second Moving Average Filter	
	**Mean Bias (95%CI)**	**R**
Q_{rest}	1.81 (1.02 – 2.59)	0.98 $p<0.001$
Q_{hyper}	-2.47 (-4.53 – -0.41)	0.98 $p<0.001$
$R_{\mu,hyper}$	4.08 (0.22 – 7.95)	0.98 $p<0.001$
CFR	-0.09 (-0.12 – -0.06)	0.96 $p<0.001$
MRR	-0.11 (-0.15 – -0.07)	0.96 $p<0.001$

"CI" = Confidence Interval, "Q_{rest}" = resting absolute flow, "Q_{hyper}" = hyperemic absolute flow, "$R_{\mu,hyper}$" = hyperemic microvascular resistance, "CFR" = Coronary Flow Reserve, "MRR" = Microvascular Resistance Reserve, "R" = Pearson's r correlation.

Supplementary Table S3. *Reproducibility data for continuous thermodilution derived indices at 2 and 10 second averages. Traces visualised with the default 2 second moving average filter for both.*

	2 Second Average					10 Second Average				
	Test 1	Test 2	R	ICC	Var (%)	Test 1	Test 2	R	ICC	Var (%)
CFR	2.73 ± 0.80	2.61 ± 0.82	0.74	0.73	17.8 ± 12.4	2.62 ± 0.72	2.57 ± 0.77	0.75	0.75	15.4 ± 11.1
MRR	3.27 ± 0.95	3.22 ± 0.97	0.72	0.72	16.5 ± 12.4	3.15 ± 0.88	3.15 ± 0.92	0.75	0.75	15.2 ± 11.2
$R_{\mu,hyper}$	408 ± 142	398 ± 129	0.88	0.88	11.9 ± 9.9	413 ± 140	402 ± 135	0.89	0.89	11.5 ± 9.8
Q_{rest}	79 ± 28	86 ± 32	0.73	0.70	20.3 ± 16.0	80 ± 27	87 ± 33	0.77	0.74	19.3 ± 14.5
Q_{hyper}	207 ± 73	216 ± 81	0.87	0.86	12.5 ± 11.1	201 ± 67	215 ± 81	0.89	0.86	12.4 ± 10.9

"Q_{rest}" = resting absolute flow, "Q_{hyper}" = hyperemic absolute flow, "$R_{\mu,hyper}$" = Hyperemic microvascular resistance, "CFR" = Coronary Flow Reserve, "MRR" = Microvascular Resistance Reserve, "R" = Pearson's r, "ICC" = Intraclass Correlation Coefficient, "Var(%)" = Variability Percentage, "Test 1" = First continuous thermodilution measurement, "Test 2" = Second continuous thermodilution measurement.

Supplementary Figure S1. *Scatter plots of 2 second average indices (x-axis), against 10 second average indices (y-axis). A =* Q_{rest}*, B =* Q_{hyper}*, C =* $R_{\mu,hyper}$*, D = CFR, E = MRR.*

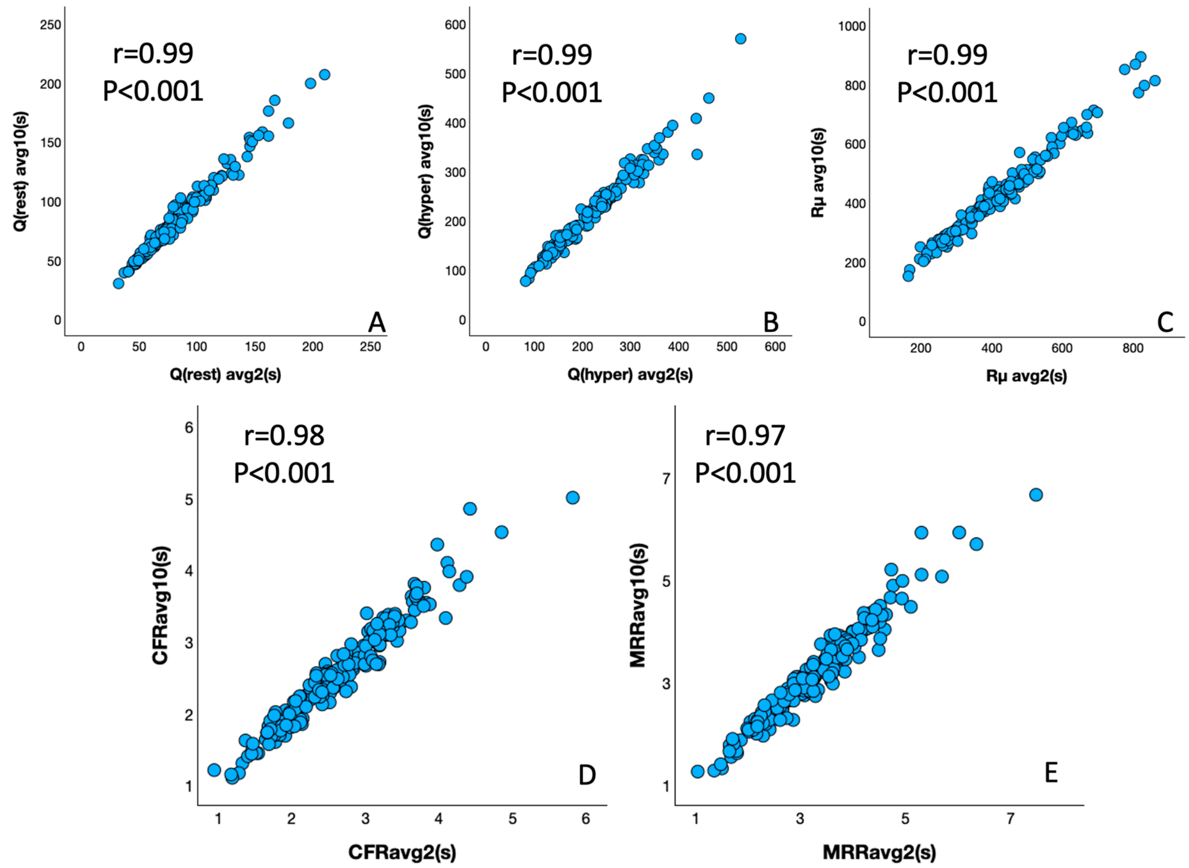

"Q" = Absolute flow, "avg2(s)" = average of 2 seconds, "avg10(s)" = Average of 10 seconds, "R_μ" = Hyperemic microvascular resistance, "CFR" = Coronary Flow Reserve, "MRR" = Microvascular Resistance Reserve.
